# Active tactile sensing of small insect force by a soft microfinger toward microfinger-insect interactions

**DOI:** 10.1038/s41598-022-21188-2

**Published:** 2022-10-10

**Authors:** Satoshi Konishi, Fuminari Mori, Yugo Kakehi, Ayano Shimizu, Fumiya Sano, Kodai Koyanagi

**Affiliations:** 1 Department of Mechanical Engineering, College of Science and Engineering, Ritsumeikan University, Kusatsu, 525-8577 Japan; 2 Graduate Course of Science and Engineering, Ritsumeikan University, Kusatsu, 525-8577 Japan; 3 Ritsumeikan Advanced Research Academy, Kyoto, 604-8520 Japan; 4 Ritsumeikan Global Innovation Research Organization, Kyoto, 604-8520 Japan

**Keywords:** Materials for devices, Soft materials, Biomedical engineering

## Abstract

Human–robot interaction technology has contributed to improving sociality for humanoid robots. At scales far from human scales, a microrobot can interact with an environment in a small world. Microsensors have been applied to measurement of forces by flying or walking insects. Meanwhile, most previous works focused on the measurement of the behavior of insects. Here, we propose microrobot-insect interactions by soft microfingers integrated with artificial muscle actuators and tactile sensors, which has been developed for a haptic teleoperation robot system. A soft pneumatic balloon actuator acts as the artificial muscle, and a flexible strain sensor using a liquid metal provides tactile sensing. Force interaction between a pill bug and the microfinger could be accomplished. The microfinger (12 mm × 3 mm × 490 μm) can move and touch an insect, and it can detect reaction force from an insect. The measured reaction force from the legs of a pill bug as a representative insect was less than 10 mN. This paper presents a microfinger as an end effector for the active sensing of reaction force from a small insect. We anticipate that our results will lead to further evaluation of small living things as well as technology development for human–environment interaction.

## Introduction

Micro electromechanical systems (MEMS) and lab-on-a-chip (LOC) technologies have integrated various functions on very small chips. Received physical and chemical signals are transformed into electrical signals and biochemical reactions can be generated and processed on a chip. Beyond detections or reactions on a chip, micromachines have potential as an intermediary tool for various interactions. A humanoid robot requires various sensors and actuators to mimic the functions of humans. Specialized and differentiated industrial robots are equipped with sensors and actuators to complete their mission. Downsized microsensors are suitable to functionalize robots without disrupting their fundamental functions. Furthermore, micromachines have potential as an intermediary tool for various interactions with a small world. Microrobots are capable of interacting with an environment in this small world, whereas humanoid robots are designed for human-robot interaction in a macro world. A combination of haptic interfaces with microrobots could even allow interaction between the small world and us.

Microsensors have been used for force measurement of small living things such as insects. The flight force of flying insects, as a typical force of insects, has been measured by various means^1–6^. Direct measurement by microsensors and image processing for motion capture have been used for force measurement. The deformation, motion and generated force of the wings of a moth were optically measured using fringe pattern projection^1^. The aerodynamic vertical force was approximately 7 mN. It is approximately 5 times stronger than the gravitational force acting on the moth (approximately 1.3 mN).

The flight forces of Drosophila were measured using a MEMS capacitive force sensor (3.6 mm × 2.1 mm × 0.5 mm) to understand flight biomechanics in Drosophila (3 mm long)^2^. The capacitive sensor was developed using a silicon on insulator (SOI) substrate to capture instantaneous flight force in real time. Drosophila samples were tethered to a sensor probe (3 mm × 50 μm × 50 μm). The total flight force was estimated at a few tens of micronewtons. The collision avoidance behavior of the locust (40 mm in body length) was investigated with simultaneous force measurements and high-speed video recording. Interesting results on the relationship between wing flapping, lift and thrust were reported^3^. The force and moment of the takeoff flight of fruitflies were analyzed using high-speed video techniques^4^. The contribution of the force of the jumping legs and the flapping wings to fly lifting was examined. The vertical force of the jumping legs (μN order) is sufficiently larger than the corresponding aerodynamic force. Social forces in the interaction by laboratory swarms of the flying midge Chironomus riparius were studied using multicamera stereoimaging and particle-tracking techniques to understand collective animal behavior^5^. The acceleration from each insect toward its nearest neighbor was measured to estimate repulsive and attractive forces in that study. The traction force by male Strepsiptera (Insecta) was measured to estimate the dependence of the surface condition of substrates^6^. The force was measured using a force sensor based on strain gauges, which were attached to the insects through a thin polymer thread. The mean values of the measured force were lower than 0.5 mN.

In addition to flight force measurement, leg forces of various insects were measured^7–10^. Measurement of the force of the leg of a walking stick insect was reported to investigate the control mechanism in positioning the joint in the legs^7^. A platform with a forcemeter was prepared in the path where stick insects walk. The median of difference between the force value at the beginning and end of the stimulus ramp was − 3.0 mN (flexion) and 6.0 mN (extension). A multiaxis piezoresistive sensor with micronewton force resolution was reported to measure the foot force of insects such as ants smaller than cockroaches^8^. The demonstrated sensor had a minimum force resolution on the order of 0.5 mN. The plant–insect interactions on leaflets were investigated by measuring attachment (traction) forces generated by beetles on various plant substrates^9^. The dorsal surface of the beetle thorax was attached to a load cell force sensor by means of hair. The measured maximal traction forces on plant surfaces differed from the force generated on glass as the control ranging from 0.5 to 11.8 mN. An array of micro force plates using strain gauges for the measurement of ground reaction forces of insect legs was reported^10^. The force resolution was 1 μN.

Not only animals but also plants generate forces to deform their shape and alter their physical characteristics. The motion of the Venus flytrap upper leaf is an example of a well-known motion generated by a plant. Measurement of forces generated by the Venus flytrap, which strikes, holds and compresses the prey, was reported^11^. A piezoelectric sensor was used for direct measurements of the average impact force of the trap together with a video camera for the determination of time constants. The impact average force between rims of two lobes in the Venus flytrap was found to be 149 mN, for example.

**Figure 1:**
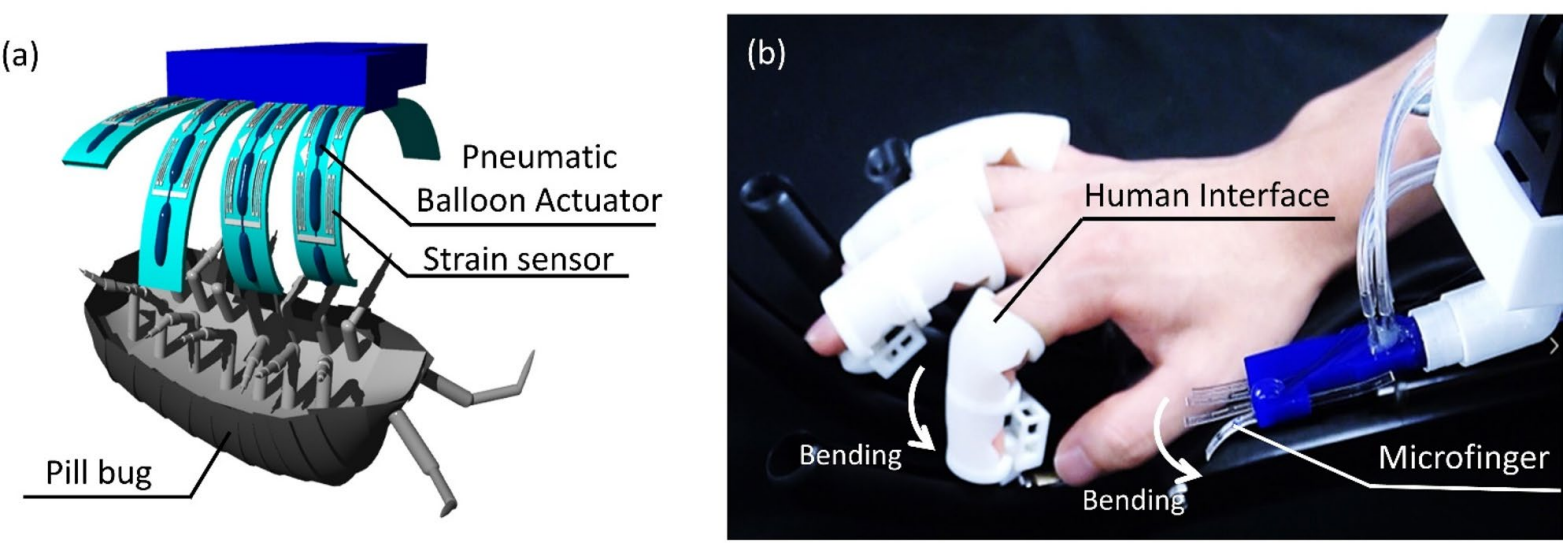
Microfinger-insect interaction. (**a**) Schematic drawing of microfinger-insect (pill bug) interaction. Shade3D Basic ver. 17.0.0 (https://shade3d.jp/en/) was used to create the images. (**b**) Photograph of a developed microhand with five microfingers. This study focuses on a single microfinger, whereas the microhand in the photograph (**b**) implies the potential of human hand-insect interactions through the haptic teleoperation robot system^19,20^.

Most previous works focused on the measurement of the behavior of insects, such as flight forces and leg forces. This paper, for the first time, presents microrobot-insect interactions by a soft microfinger integrated with an artificial muscle actuator and tactile strain sensor as shown in Fig. 1a. A microfinger can apply force to an objective insect and stimulate the insect. The artificial muscle actuator for a microfinger, which is a pneumatic balloon actuator (PBA) made of polymer, is soft and safe enough to interact with insects gently^12,13^. Manipulation robot systems with microfingers have been developed using PBA for object-grasping motion^12^. A minuscule fish roe was successfully manipulated. Furthermore, microfingers for cellular aggregate manipulation have been developed^13^. A spherical human mesenchymal stem cell (hMSC) aggregate (φ200 μm) was pinched and released on a microwell plate. Our study, in addition to artificial muscle microactuators, incorporates the integration of tactile sensors into a microfinger. A flexile temperature sensor was integrated into a microfinger for temperature sensing functionality^14^. Several types of strain sensors were studied for motion detection of a microfinger. Recently, a strain sensor using a microchannel filled with liquid metal (Galinstan) was developed for PBA^15–17^. The liquid metal strain sensor is a resistive type and its gauge factor is reported approximately 1^15^. The strain sensor can be fabricated by filling liquid metal into microchannels. This study takes advantage of the common channel structures for both sensor and pneumatic balloon actuator. This study presents that the liquid metal strain sensor integrated into the microfinger can detect the reaction force from an insect. Therefore, the microfinger enables active force sensing against living insects. Previously, bilateral mechanical scaling instrument was developed and applied to interactions between an insect by Mohand Ousaid et al.^18^. The instrument system was composed of an active probe and hand interfaces for the bilateral interaction. They used an insect leg for probing droplets of water. We also have developed and reported a haptic teleoperation robot system composed of a slave microfinger and a master interface device for an operator^19,20^. This paper presents a microfinger as a microscopic end effector for the active sensing of reaction force from an insect, which has the potential of microfinger-insect interactions in the small world in combination with the interaction system, such as a bilateral control system^18^ and a haptic teleoperation system^19,20^.

## Results and discussion

Figure 1a illustrates an image of microfinger-insect interactions. A pill bug is illustrated as a representative insect in Fig. 1a. Microfingers move and apply force to a pill bug lying on its back. The pill bug reacts to the force and pushes back on the microfingers. The microfingers can detect the reaction force from the pill bug by their strain sensors. Active sensing by the microfingers enables microfinger-insect interactions. Figure 1b shows a photograph of a developed microhand with five microfingers. This study focuses on a single microfinger integrated with artificial muscle microactuators (PBAs) and a tactile sensor.

### Microfingers for active force sensing of a pill bug

**Figure 2:**
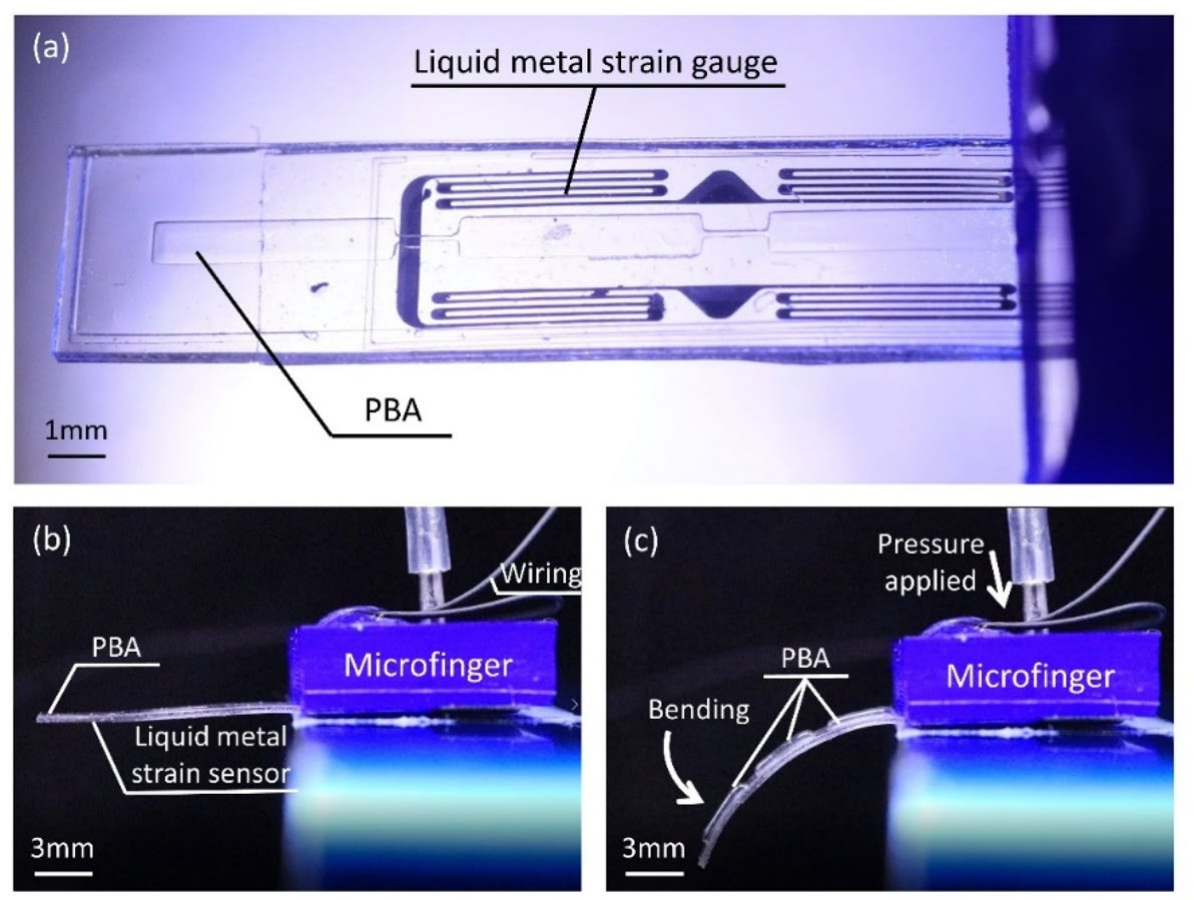
Developed microfinger (12 mm long, 3 mm wide, 490 μm thick) for active force sensing of a pill bug. (**a**) Photograph of a top view of microfinger. (**b**) Side view of an initial state of a microfinger. (**c**) Side view of bending motion of a microfinger.

Figure 2 shows photographs of a developed microfinger for active force sensing of a pill bug. Figures 2a–c show photographs of a top view and side views of an initial state and bending motion of a microfinger, respectively. The developed microfinger was 12 mm long, 3 mm wide, and 490 μm thick, and was designed for a pill bug. The body length of the sampled pill bugs ranged from 8 to 16 mm. The microfinger was made of polydimethylsiloxane (PDMS) and integrated with a PBA and a strain sensor using liquid metal^15–17^. The resistive strain sensor used an electrical resistor composed of liquid metal filled in a microchannel which was fabricated together with a structure of pneumatic balloon actuator for the microfinger.

**Figure 3:**
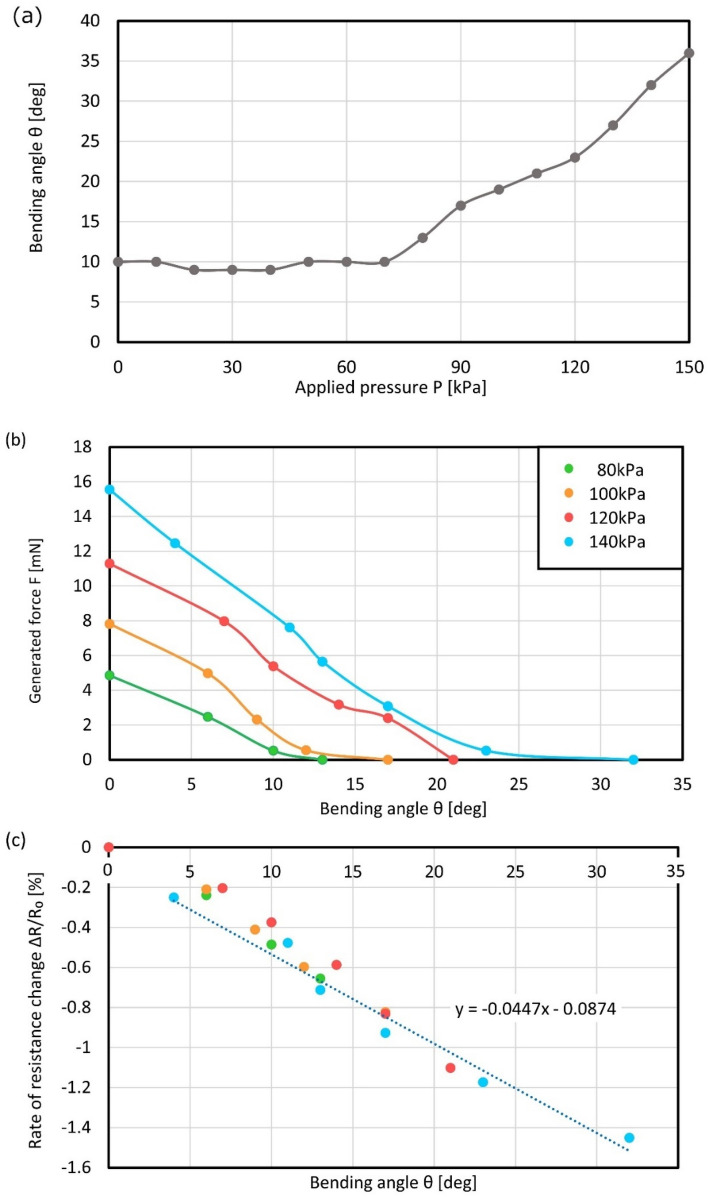
Characterization results of the bending motion of a microfinger and the force generated by a microfinger. (**a**) Characteristics of bending motion by a microfinger. (**b**) Output characteristics of a microfinger in terms of bending angle and generated force in accordance with applied pressure. (**c**) Characteristics of a flexible strain sensor for a microfinger. Data processing software (BenchVue, Keysight, ver. 3.1.1602.19, https://www.keysight.com/jp/ja/lib/software-detail/computer-software/benchvue-installation-wizard-2417463.html) was used for signal processing.

Figures 3a–c show the characterization results of the bending motion of a microfinger and the force generated by a microfinger. The relationship between the applied pressure and bending angle is shown in Fig. 3a. The bending angle increased in accordance with the applied at pressure higher than 70 kPa. Figure 3b summarizes the output characteristics of a microfinger in terms of both bending angle and generated force in accordance with the applied pressure. Figure 3c shows the characteristics of a flexible strain sensor for a microfinger. A strain sensor used the electrical resistance of liquid metal. Galinstan (eutectic gallium indium stannum) as a typical liquid metal was filled into a microchannel for a flexible strain sensor with good compatibility with PBA using microchannels for pressure supply. A strain sensor showed linear characteristics against the bending angle. The performance of PBA with a strain sensor for a microfinger was further verified through 100 times repeated driving test. The initial bending angle of PBA was set at 42 deg. The bending angle resulted in 43 deg after 100 times actuation. The relative resistance change was initially − 1.013% and resulted in − 1.039% after 100 times bending motion. Although characteristics of the microfinger is supposed to be calibrated each time, the results show sufficient performance.

**Figure 4:**
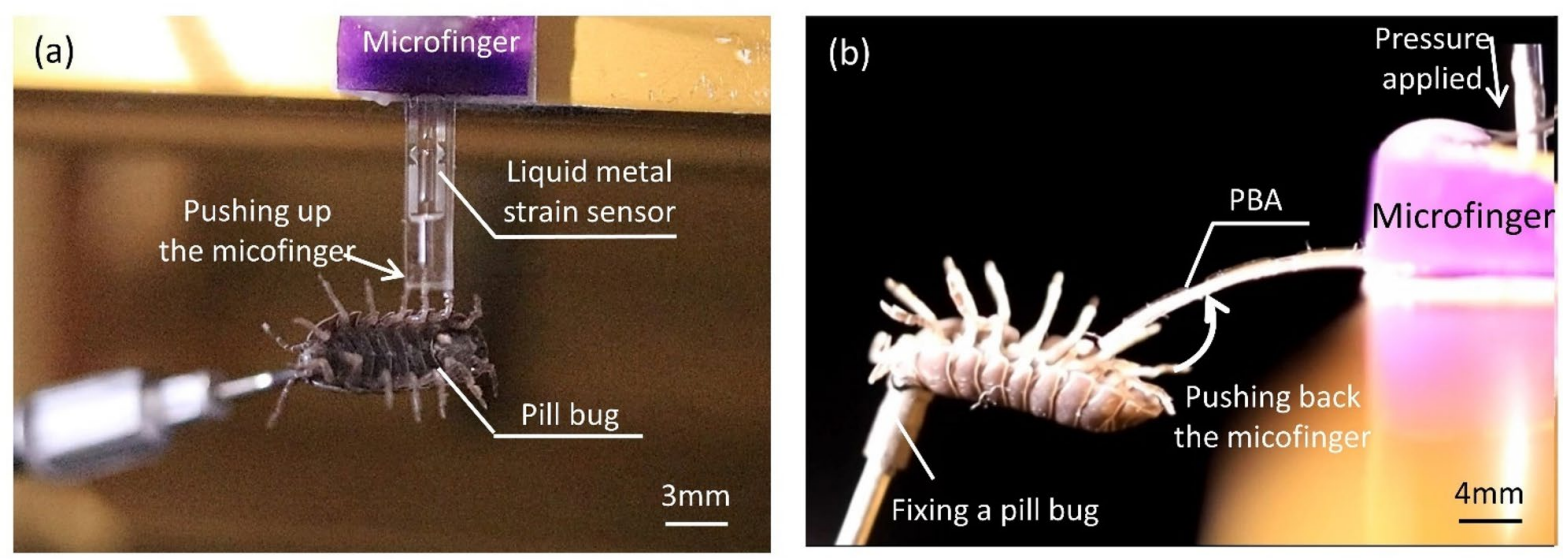
Force measurement of a pill bug by a microfinger. (**a**) Active sensing of leg force. (**b**) Active sensing of abdominal force. The pill bug was immobilized by vacuum tweezer device. The attitude angle of a pill bug was adjusted for optimum contacts against the leg and body. The generated force by a microfinger was 15.6 mN at 140 kPa.

This study aimed to measure both leg force and abdominal force of a pill bug. The applied pressure for force measurement experiments was set at 140 kPa, which could generate 15.6 mN. Figures 4a,b show photographs of active sensing against a pill bug lying on its back. Supplemental Videos S1 and S2 show more details of the measurement experiments, especially the motion of a pill bug. Active sensing by a microfinger was used to measure the forces of a pill bug. A pill bug was immobilized by applying suction to its back. A vacuum tweezer device was arranged and used as an insect fixing base. Figure 4a shows active sensing of a leg force of a pill bug, whereas Fig. 4b shows active sensing of an abdominal force. The attitude angle of a pill bug was adjusted according to requirements for optimum contacts against the leg and body, as shown in Fig. 4. The angular position of a suction tool fixing the back of a pill bug was controlled. A pill bug was set at sideward posture in the leg force measurement, whereas it was adjusted at upward posture in the abdominal force measurement.

### Force measurement of a pill bug by a microfinger

**Figure 5:**
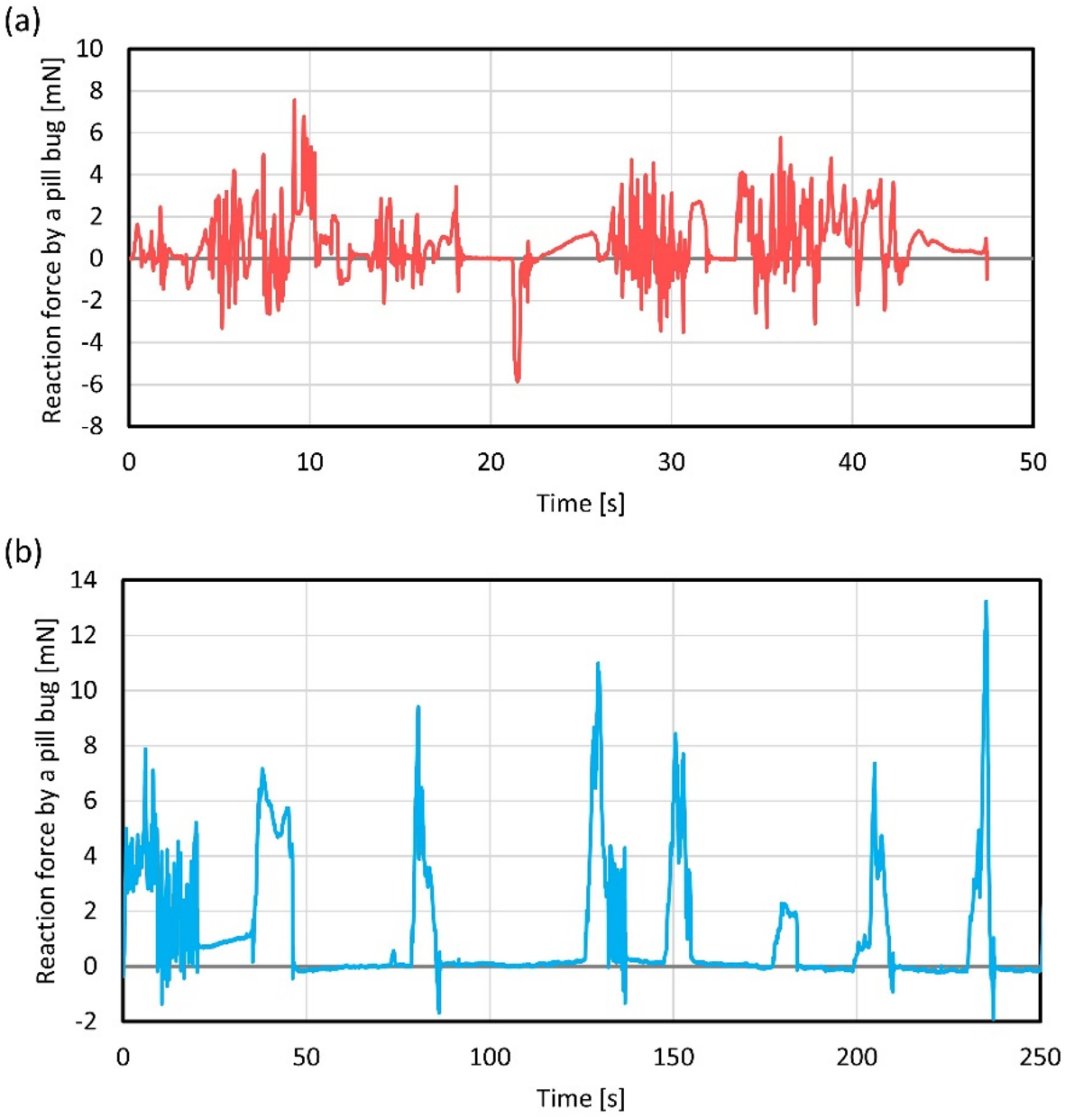
Force measurement of a pill bug through active sensing by a microfinger. (**a**) Measured leg force in accordance with time. (**b**) Measured abdominal force in accordance with time. The measured leg force was less than 10 mN, whereas abdominal force exceeded 10 mN. The frequency of each motion showed different tendency.

Figure 5 shows the measured forces of a pill bug through active sensing by a microfinger. Typical case examples among measured data are demonstrated in Fig. 5. Figures 5a,b show the measured leg and abdominal forces over time, respectively. The measured leg force was less than 10 mN as shown in Fig. 5a. The leg motion happened at approximately 0.3 Hz. According to supplemental Video S1, detected reaction showed higher frequency (1.6 Hz) because a plurality of legs pushed the microfinger alternately. Measured abdominal force was larger than leg force and exceeded 10 mN as shown in Fig. 5b. The frequency of abdominal motion in Fig. 5b was lower than that of leg motion and was estimated to be approximately 0.03 Hz. The frequency of leg motion was much higher than that of abdominal motion. Supplemental Videos S1 and S2 show differences in leg and abdominal motions. A larger displacement amount of motion pushing back a microfinger tended to show a larger force.

The detected information can be utilized for the haptic teleoperation robot system as presented in Fig. 1b, which uses the microfinger as a slave component in combination with a master interface device for an operator^16,17^. The system is capable of presenting touch sense acquired by the microfinger-insect interaction thorough the haptic teleoperation robot system.

### Dependence of force and weight of a pill bug

**Figure 6:**
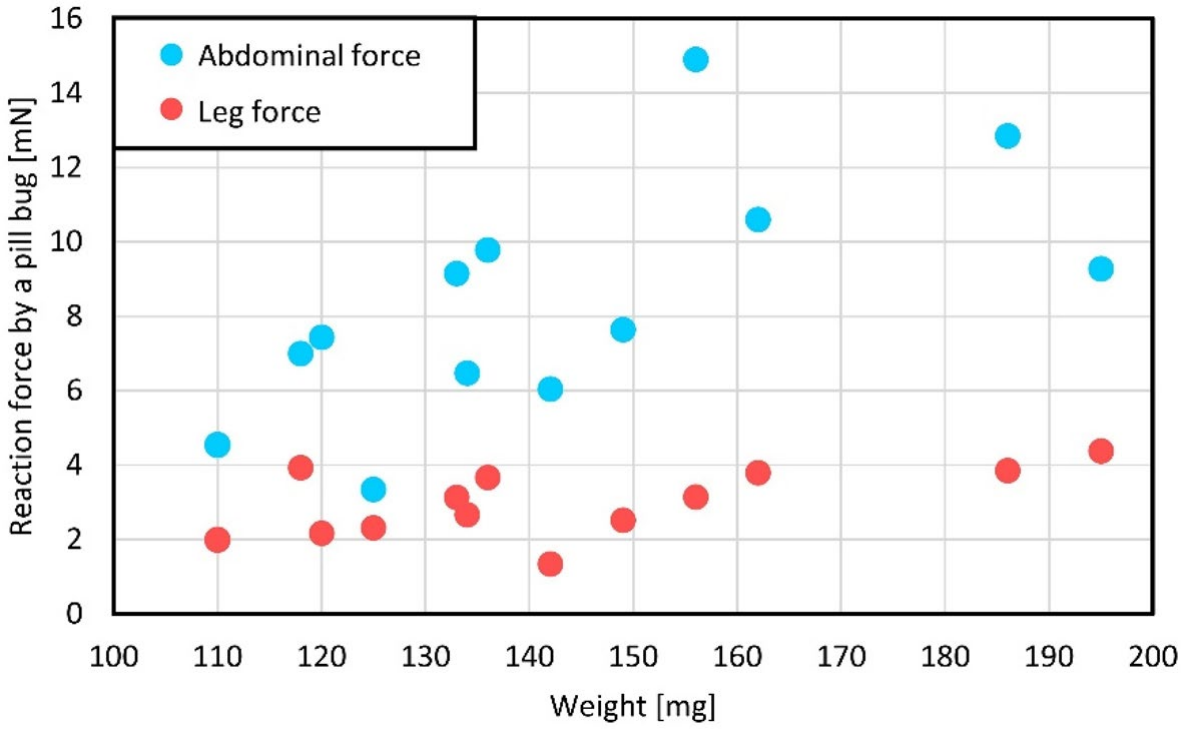
Leg forces and abdominal forces of pill bugs in relation to their weight. The weight distribution of the collected pill bugs was between 100 and 200 mg. The tendency of increasing the force in relation to the wight was examined.

Forces by pill bugs were estimated from the point of view of weight dependence. Thirteen pill bugs were collected for the evaluation. Figure 6 reports leg forces and abdominal forces of pill bugs in relation to their weight. The weight of the pill bugs was also measured during the force measurement. The weight distribution of the collected pill bugs was between 100 and 200 mg.

The abdominal force of pill bugs weighing under 160 mg tended to increase depending on the weight of the pill bug. A pill bug of 158 mg in weight generated a maximum abdominal force of 15 mN according to a linear relationship. However, abdominal force generated by pill bugs whose weight was over 160 mg showed different tendencies. Regarding the sampled pill bugs in our experiments, pill bugs weighing over 160 mg did not show a larger force than those weighing under 160 mg, contrary to the expected linear relationship between weight and abdominal force. The tendency of increasing the force was not as prominent in the case of leg force. The leg force was less than 10 mN for the collected pill bugs. As references, leg forces and abdominal forces of other seven pill bugs were measured by a commercialized load cell (LVS-5GA, Kyowa Electronic Instruments Co.). The average of weight, leg force, and abdominal force of seven pill bugs were 155 mg, 2.1 mN, and 9.0 mN, respectively. We can see that measured results in Fig. 6 were in the similar range to the reference data measured by a load cell.

## Materials and methods

### Fabrication of a microfinger integrated with PBAs and a strain sensor

**Figure 7:**
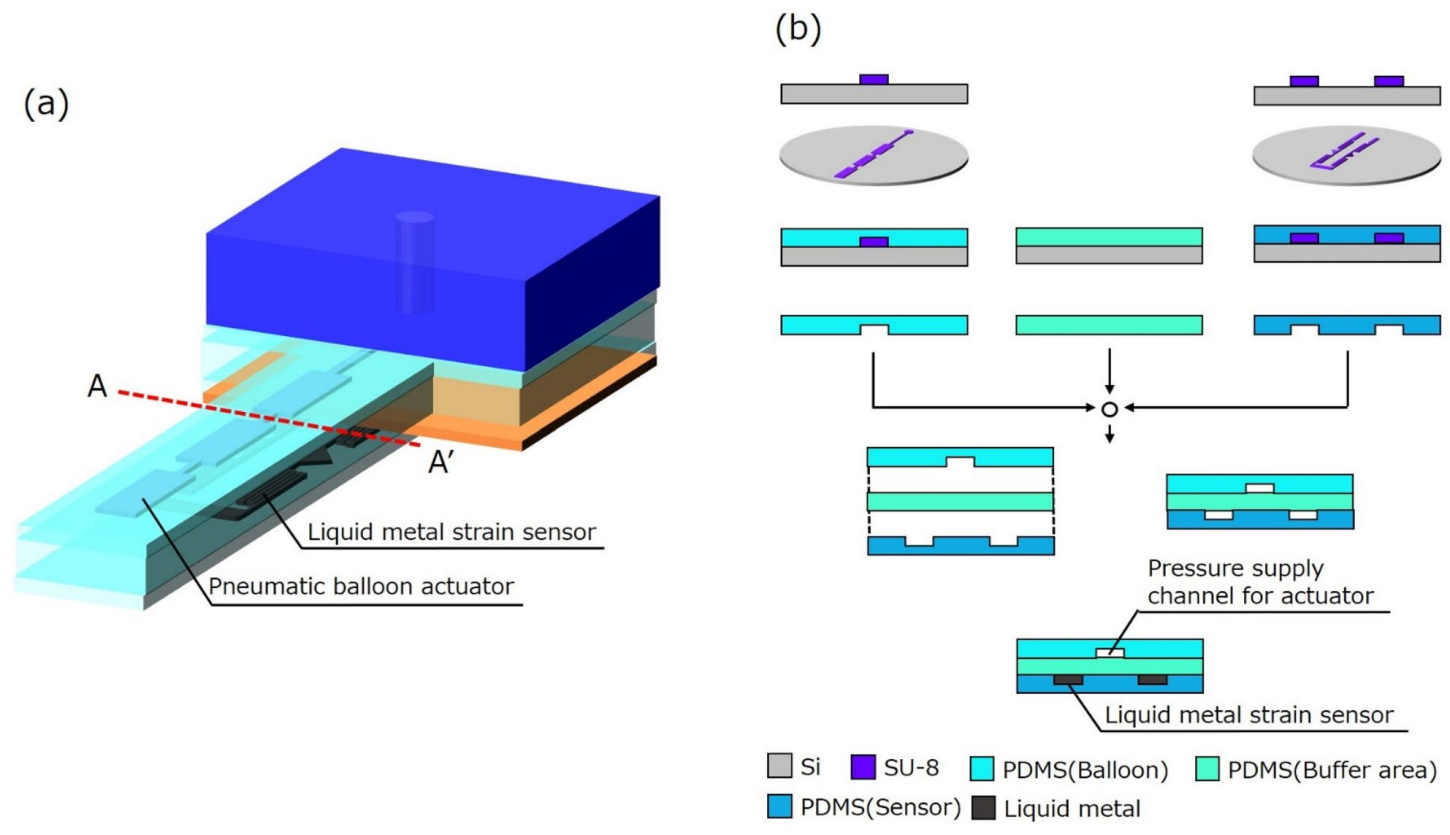
Fabrication process of the microfinger integrated with PBA and a liquid metal-based sensor. (**a**) Schematic image of the microfinger. (**b**) Fabrication process using cross sections along A–A’ in Fig. 7a. Three PDMS layers are bonded to form spaces for microchannels. The resistive strain sensor is completed by filling liquid metal in the microchannels.

A microfinger (12 mm × 3 mm × 490 μm) was integrated with PBAs and a strain sensor. Figure 7 shows the fabrication process which was common with previously reported process in^15–17^. PDMS layers were prepared by molding PDMS (Silpot 184, Dow Corning Inc.) on a photoresist (SU-8) mold on a Si substrate. Microchannels for both PBA and a liquid metal-based sensor were formed by bonding three PDMS films. The microchannel was 50 μm wide and 50 μm high. The width of balloon region was 800 μm. Bonded PDMS films were equipped with PDMS interconnections. Galinstan (eutectic gallium indium stannum, Zairyo-ya.com) was injected into microchannels for a sensor and wired through an interconnection.

### Experimental setup for device characterization

The bending angle and generated force of the microfinger were evaluated according to the applied pressure. The pressure was controlled and supplied by a combination of a compressor (AS4P-6, Kobe Steel, Ltd.) and electropneumatic regulator (ITV0050-3MS, SMC Corporation). The bending angle was estimated through captured images by digital camera (EOS Kiss X9i, Canon Inc.). A load cell (LVS-5GA, Kyowa Electronic Instruments Co.) was used to measure the generated force. Detected signal by a load cell was amplified by a strain amplifier (DPM-911B, Kyowa Electronic Instruments Co.). A digital multimeter (34460A, Keysight Tech.) equipped with data processing software (BenchVue, Keysight, ver. 3.1.1602.19) was used for signal processing of both acquired force signal and resistive change of a liquid metal strain sensor.

### Experimental setup for measurement of reaction force from an insect

A suction tool was used to fix a pill bug by sucking its back. A vacuum tweezer device (TWEEZER-VAC™, VIRTUAL) was utilized as an insect fixing base as shown in Fig. 4. The pill bug was immobilized by a suction pump (ASPIRATOR Labo Helper, TOKYO M.I. COMPANY, INC.). The microfinger was initially positioned by using a stage (lab jack, 105, Julabo Japan) and was approached and made contact with a pill bug by actuating its artificial muscle. The electrical resistance of the liquid metal-based strain sensor was measured by a digital multimeter (34460A, Keysight Tech.) in combination with data processing software (BenchVue, Keysight, ver. 3.1.1602.19).

### Derivation method for reaction force from an insect

**Figure 8:**
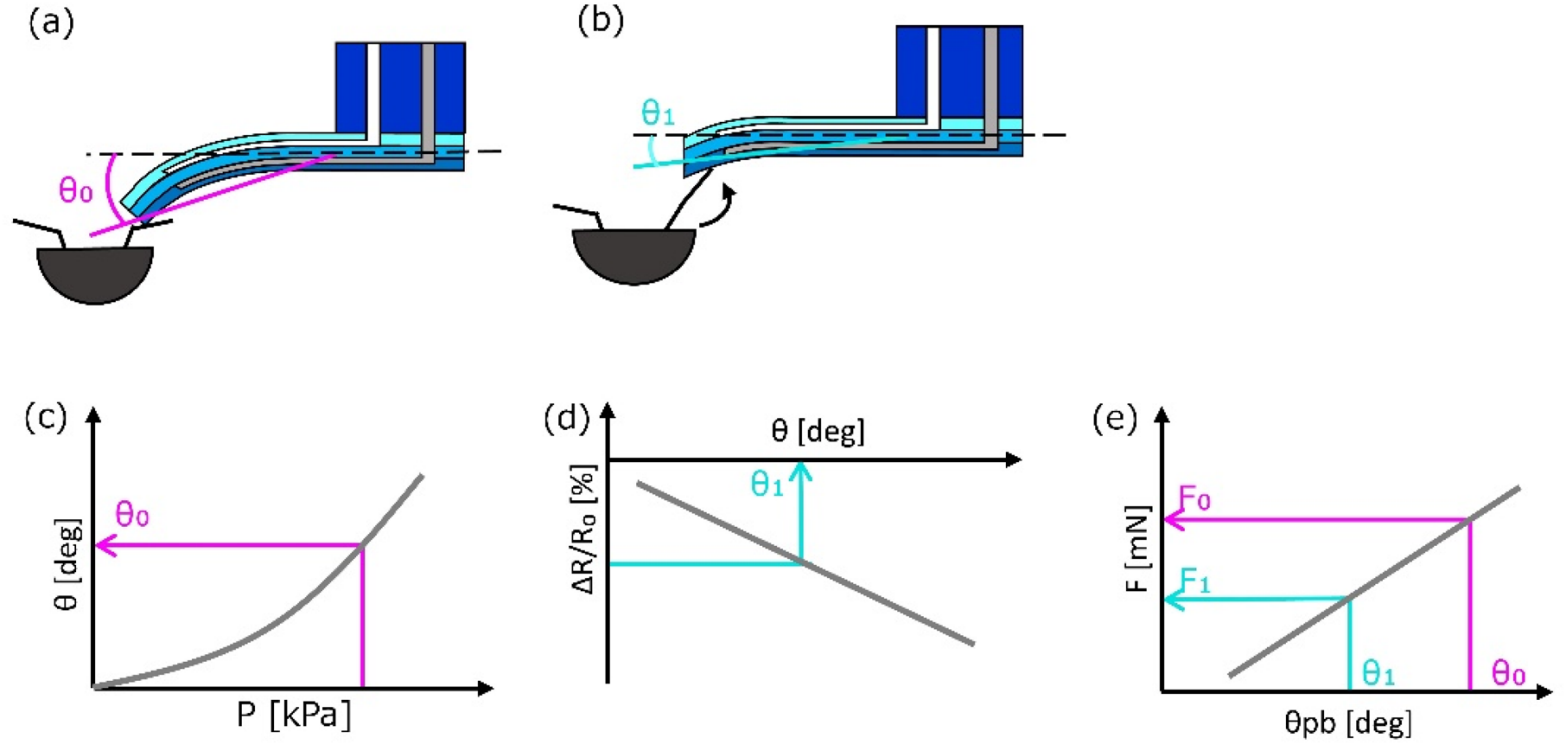
Force measurement principle for active sensing by a microfinger. (**a**) Microfinger approaching a pill bug. (**b**) Interaction with a pill bug. (**c**) Expected force corresponding to the applied pressure based on the obtained relationship. (**d**) Bending angle θ_1_ obtained by the sensor signal. (**e**) Reaction force calculated using a difference of corresponding forces to θ_0_ and θ_1_.

**Figure 9:**
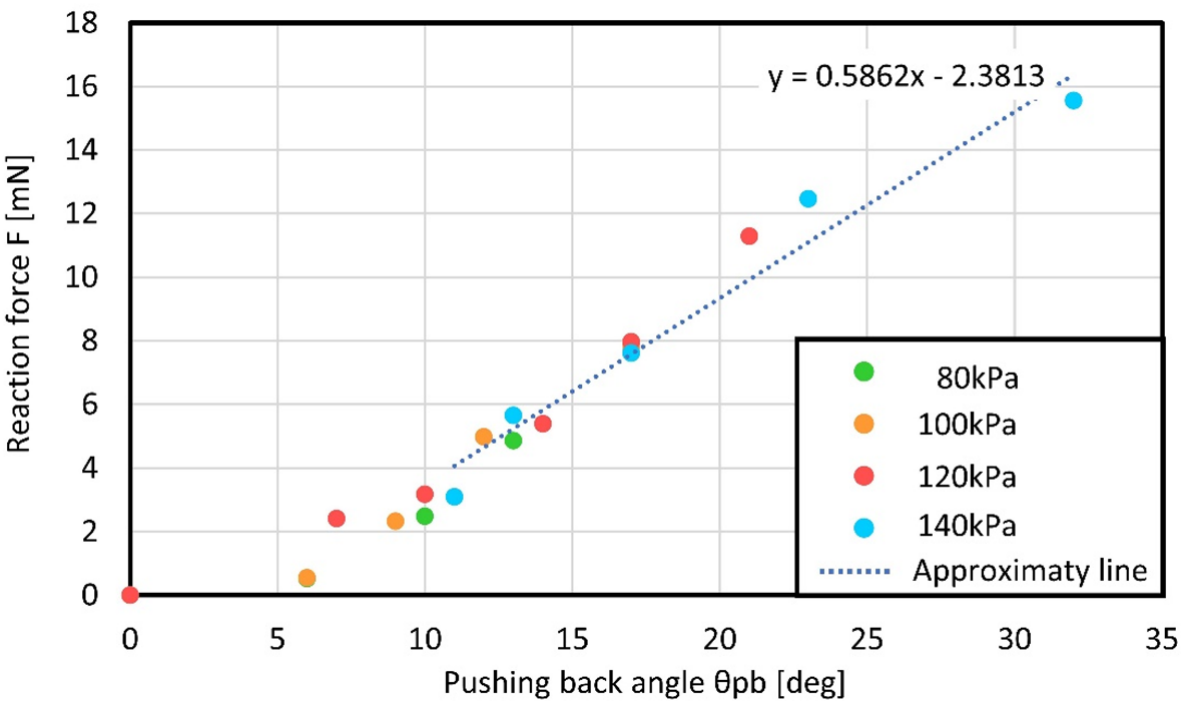
Relationship between pushing back angle θ_pb_ (θ_pb_ = θ_0_–θ_1_) and reaction force F, which is obtained through the calculation steps from (**c**) to (**e**).

Figure 8 explains the derivation method for the reaction force through active sensing by a microfinger. Figure 8a,b illustrates an interaction between the microfinger and a pill bug. A microfinger touches when it bends at θ_0_ as shown in Fig. 8a. The bending angle of a microfinger becomes θ_1_ when a pill bug pushes back a microfinger (Fig. 8b). Figure 8c–e show sequential steps of the derivation method for the reaction force from a pill bug. Figure 8c,d correspond to the characteristics in Fig. 3a,c, respectively. First, the expected force corresponding to the applied pressure is obtained through the obtained relationship in advance (Fig. 8c). We set applied pressure at 140 kPa in our experiments. Next, the current bending angle θ_1_ is evaluated using the signal detected by a strain sensor in a microfinger (Fig. 8d). The relative resistance change ΔR/R0 is measured and converted into bending angle θ. Finally, a reaction force is calculated using a difference of corresponding forces to θ_0_ and θ_1_, as shown in Fig. 8e. As a result of the calculation steps from (c) to (e), the relationship between pushing buck angle θ_pb_ (θ_pb_ = θ_0_–θ_1_) and reaction force F can be obtained as shown in Fig. 9, which is available for the touch sense presentation.

## Supplementary Information

Supplementary Information 1.

Supplementary Video 1.

Supplementary Video 2.

## Data Availability

All data generated or analyzed during this study are included in this published article.
