# Supplementary material for: Active tactile sensing of small insect force by a soft microfinger toward microfinger-insect interactions
Source: Sci Rep. 2022 Oct 10;12:16963. doi: 10.1038/s41598-022-21188-2 (PMC9550801; doi:10.1038/s41598-022-21188-2)
Supplement: Supplementary file 1 — Supplementary Information 1. [file 41598_2022_21188_MOESM1_ESM.pdf]

Active Tactile Sensing of Small Insect Force by a Soft Microfinger toward Microfinger-Insect Interactions

Satoshi Konishi[1,2,3,4*], Fuminari Mori[2], Yugo Kakehi[2], Ayano Shimizu[2], Fumiya Sano[2], Kodai Koyanagi[2]

[1]Department of Mechanical Engineering, College of Science and Engineering, Ritsumeikan University, Kusatsu, 525-8577, Japan

[2] Graduate Course of Science and Engineering, Ritsumeikan University, Kusatsu, 525-8577, Japan

[3] Ritsumeikan Advanced Research Academy, Kyoto 604-8520, Japan

[4] Ritsumeikan Global Innovation Research Organization, Kyoto 604-8520, Japan

[*]konishi@se.ritsumei.ac.jp

Supplementary Movie Legend

Movie S1. Leg force measurement of a pill bug through active sensing by a microfinger. The pill bug was immobilized by vacuum tweezer device with a suction pump. The attitude angle of a pill bug was adjusted for the optimum state for interaction between legs.

Movie S2. Abdominal force measurement of a pill bug through active sensing by a microfinger. The attitude angle of a pill bug was adjusted for the optimum interaction between a body.
